# Sodium dependent glucose transporter (SGLT) 1 / 2 - elucidating inhibitor SAR and selectivity using homology modelling and 3D QSAR studies

**DOI:** 10.1186/1758-2946-4-S1-P41

**Published:** 2012-05-01

**Authors:** Susann Vorberg, Ina Koch, Christian Buning

**Affiliations:** 1 Sanofi-Aventis Deutschland GmbH, Industriepark Höchst, Building G838, 65926 Frankfurt a.M., Germany; 2 University Frankfurt, Institute for Computer Science, Department for Molecular Bioinformatics, 60325 Frankfurt a.M., Germany

## 

Inhibiting sodium-dependent glucose transporters (SGLTs) has been proposed as a new therapy for the treatment of diabetes [1]. SGLT2 as the most prominent member of this family is mainly expressed in the kidney and responsible for the reabsorption of the vast majority of the filtered glucose. This key role in the blood glucose homeostasis makes SGLT2 a promising target which has been clearly underlined by the results of preclinical and clinical studies. Therapeutic goals of SGLT2 inhibition are reduced plasma glucose levels and weight loss. In conjunction with the therapeutic benefits fewer side effects are expected than observed with other known diabetes drugs. Potential side effects in case of SGLT2 inhibition are expected to be mediated by a lack of selectivity towards SGLT1 that is mainly expressed in the intestine and responsible for glucose- and galactose absorption from food sources. Therefore, inhibition of SGLT1 leads to glucose- and galactose malabsorption, dehydration, and diarrhoea.

Consequently, any drug discovery project aiming for promising SGLT2 inhibitors has to take into account significant selectivity towards SGLT1. In order to describe the selectivity on a structural basis we extensively used molecular modelling techniques since no x-ray structural data is available for neither SGLT1 nor SGLT2. For both transporters homology models were generated using the published x-ray structure from vSGLT (PDB-code 3DH4) [2]. Structure based alignments of published SGLT2 inhibitors including selectivity data for SGLT1 were followed by 3D-QSAR studies utilizing CoMFA fields. Given a detailed analysis of the inhibitor SAR for both transporters the two structural models of SGLT1 and SGLT2 were compared leading to the identification of relevant differences and selectivity hot spots. In the future, the presented models could serve as a basis for the identification of new potent SGLT2 inhibitors that are selective towards SGLT1, too.
